# Twenty years population-based trends in prevalence, awareness, treatment, and control of hypertension in Geneva, Switzerland

**DOI:** 10.1016/j.pmedr.2025.103055

**Published:** 2025-04-01

**Authors:** Mayssam Nehme, Anshu Uppal, Ophelia Zimmerman, Julien Lamour, Shannon Mechoullam, Idris Guessous

**Affiliations:** a Division of Primary Care Medicine, Geneva University Hospitals, Geneva, Switzerland; b Faculty of Medicine, University of Geneva, Geneva, Switzerland

**Keywords:** Hypertension, Epidemiology, Risk factors, Prevalence, Awareness, Cardiovascular disease, Primary care, Year-trends study

## Abstract

**Objective:**

Hypertension is a leading cause of cardiovascular disease and affects about 1.3 billion adults worldwide. Despite interventions, awareness and control remain suboptimal and might have worsened due to the COVID-19 pandemic. This population-based study examines 20-year trends in hypertension prevalence, awareness, treatment, and control in Geneva, Switzerland (2005–2023).

**Methods:**

This is a year-trends population-based study (Bus Sante) ongoing in Geneva, Switzerland. Data collected in this study were between 2005 and 2023. Hypertension trends and prevalence were stratified by sex, age, education, and income. Multivariable regression models adjusted for sociodemographic and health factors identified determinants of outcomes.

**Results:**

Overall, 11,278 individuals participated. Hypertension prevalence decreased from 38.9 % to 35.2 %, with greater reductions in individuals with primary education (−6.1 %) and low income (−6.1 %). Awareness remained stable with time. Uncontrolled hypertension decreased (44.9 % to 42.2 %, *p* = 0.01), with improvements in lower socioeconomic groups, and individuals with diabetes. Older women were more likely to have untreated (+16.1 %) and uncontrolled hypertension, while younger men exhibited higher unawareness rates (57.7 %). Having a doctor visit in the past 12 months was not associated with increased awareness.

**Conclusions:**

Hypertension prevalence and control improved overall, with reduced socioeconomic disparities. However, some groups remain at risk and primary care is essential for better screening, awareness, treatment, and control of hypertension.

## Highlights

• Hypertension prevalence decreased from 39.4 % to 35.1 %.
• The greatest reductions were in individuals with primary education and low income.
• Regular doctor visits did not lead to increased awareness of hypertension.

## 1 Introduction

Hypertension is one of the most prevalent chronic diseases, and a main cause of disease burden worldwide (NCD Risk Factor Collaboration (NCD-RisC), 2021). In a recent study, the prevalence of hypertension was shown to double between 1990 and 2019 worldwide (NCD Risk Factor Collaboration (NCD-RisC), 2021). An estimated 1.3 billion adults aged 30–79 years worldwide have hypertension, and two-thirds live in low- and middle-income countries (World Health Organization, 2023). In the United States, a study showed that nearly half of adults suffered from hypertension (48.1 %) between 2003 and 2014, and several initiatives were launched to better diagnose and control hypertension. Based on NHANES data, the age-adjusted prevalence of hypertension was highest in 1999–2000 (47.9 %), with a decrease between 2009 and 2014 (43.0 %) and a slight increase in 2017–2020 (44.7 %) (Jaeger et al., 2023). Additionally, rates of awareness improved (Guo et al., 2012), however these numbers fluctuate with time (Egan et al., 2021) with the latest results showing that the COVID-19 pandemic exacerbated the decline in hypertension awareness and control due to disruptions in routine healthcare (Abdalla et al., 2023). An estimated 46 % of adults with hypertension are unaware of having hypertension and less than half (42 %) are diagnosed and treated (World Health Organization, 2023). Among those treated, four out of five adults worldwide still have uncontrolled hypertension (World Health Organization, 2023). In the United States, recent results showed that 66.3 % of individuals qualifying for primary or secondary prevention of hypertension did not know they may have hypertension, and were not being treated (Heaton et al., 2024), and 55.2 % had uncontrolled blood pressure (Heaton et al., 2024) according to the 2017 American College of Cardiology ACC/AHA high blood pressure guidelines (Whelton et al., 2017).

Hypertension prevalence varies by age, sex (Danon-Hersch et al., 2009; Stringhini et al., 2012), educational level (de Mestral et al., 2024) and household income (de Mestral et al., 2024) and is higher in socioeconomically disadvantaged groups (de Mestral et al., 2024). Despite some improvements in hypertension awareness, treatment and control over time, socioeconomically-driven differences persist (Foti et al., 2019), continuing to affect disadvantaged populations. Risk factors associated with hypertension also include increased body-mass index (Tang et al., 2022), dyslipidemia (Tang et al., 2022), and diabetes (Petrie et al., 2018). The risk of untreated or uncontrolled hypertension are age, race, sex (Heaton et al., 2024; Fryar et al., 2017), smoking, obesity (Wei et al., 2021), and alcohol (Zhao et al., 2020) among others. Educational inequalities are also more pronounced in women compared to men (Stringhini et al., 2012) with hypertension.

Similar results are seen in Europe; however, data are scarcer. For example, in Geneva, Switzerland hypertension prevalence was stable between 1999 and 2009 (Guessous et al., 2012). A study from the NCD risk factor collaboration group showed that the prevalence of hypertension for both men and women in Switzerland was one of the lowest compared to other countries (NCD Risk Factor Collaboration (NCD-RisC), 2021). However, it is important to note, while overall prevalence is low, trends also differ by socioeconomic risk factors. Recent results by our study group showed that relative and absolute inequalities (*via* calculations of relative index of inequality and slope of index of inequality) remained high for hypertension, dyslipidemia, and sedentary behavior, potentially further contributing to hypertension comorbidity and disease burden (de Mestral et al., 2024). Additionally, In Switzerland, more than half of hypertensive individuals remained untreated or uncontrolled between 2004 and 2009 (Guessous et al., 2012), highlighting the need to further explore this disease even in developed countries like Switzerland.

Additionally, the post-pandemic landscape might show differences in health behaviors, access to care (Abdalla et al., 2023; Ruth et al., 2021), and an increasing health debt with an increase in post-COVID chronic conditions (Xie et al., 2022; Angeli et al., 2024). Updated and detailed group-specific trends are needed for a precise and targeted approach to population health. In this study, we evaluate the 20-year trends in hypertension prevalence, awareness, treatment, and control, offering a first look after the COVID-19 pandemic, in a population-based study in Geneva, Switzerland, while identifying potential risk factors.

## 2 Methods

### 2.1 Participants

Data were collected through the Bus Santé, a cross-sectional population-based health survey in Geneva Canton, Switzerland. Since 1992, on a yearly basis except during the 2020–2022 COVID-19 pandemic, a representative random sample of 1000 participants is recruited from the general population in Geneva, stratified according to age and sex. Eligible individuals are identified through a standardized process based on an annual residential list provided by the local government. Subjects are first contacted *via* a mailed invitation letter, followed by up to seven phone calls and two more letters. Those who are not reached after these attempts are replaced using the same procedure. Initially, the age range for participants was 35–74 years, but this was expanded to 20–74 years in 2011. For this study, only participants who were 35–74 years old were included to allow for time trends between 2005 and 2023. Participation rate was 49 %. The Bus Santé study has been approved by the Ethics Committee of the Geneva University Hospitals, and all included participants signed written informed consent (IRB00003116 1992; 2010 and CCER 2022–01544 2022; 2024). All procedures were performed in compliance with the relevant laws and institutional guidelines.

### 2.2 Data collection

Participants complete a total of five surveys, covering risk factors for major lifestyle-related chronic conditions, sociodemographic characteristics, education, occupation, income, dietary intake and physical activity using standardized questionnaires (Marques-Vidal et al., 2017; Bernstein et al., 1998). A medical visit with a research nurse is then organized to complete the physical assessment and blood tests. During the medical visit, height (precision 1 cm), weight (precision 0.5 kg, without shoes and using a medical scale), and waist-to-hip ratio are measured. After a 10-min rest, three blood pressure measurements are taken in the sitting position, using a validated automated oscillometric sphygmomanometer (OmronH Hem-907, Matsusaka, Japan). A mean of the three measurements is then calculated for systolic and diastolic blood pressure. During the medical visit, fasting blood samples including glucose, total and high-density lipoprotein (HDL), plasma cholesterol and triglycerides are collected and then analyzed using commercially available enzymatic kits (Bayer Technicon Diagnostics).

### 2.3 Measurements

Sociodemographic variables included sex (male, female), age (35–49; 50–64; 65 and above), education (primary, secondary, tertiary), income (low, middle-low, middle-high, high). Health variables included smoking status (never smoker, ex-smoker, current smoker), body-mass index BMI (normal, underweight, overweight, obese), diabetes (yes, no), dyslipidemia (yes, no), and doctor visit in the past 12 months (yes, no). The presence of diabetes was defined based on participants answering yes to the question of having diabetes, or having been diagnosed with the condition, or if they were taking antidiabetic medications in their treatment list. The presence of dyslipidemia was defined based on participants answering yes to the question of having dyslipidemia or high cholesterol, or having been diagnosed with the condition, or if they were taking cholesterol medications in their treatment list. Hypertension was defined as mean systolic and/or diastolic blood pressure ≥140/90 mmHg, self-reported hypertension, or self-reported use of anti-hypertensive treatment. Hypertension unawareness was defined as mean systolic and/or diastolic blood pressure ≥140/90 mmHg in individuals who answered no to the question “Have you ever been told that you had high blood pressure?”. Untreated hypertension was defined as individuals who reported having been diagnosed with hypertension and who were not taking anti-hypertensive treatment. Uncontrolled hypertension was defined as individuals who were diagnosed with hypertension and taking anti-hypertensive treatment, and who had a mean systolic and/or diastolic blood pressure ≥140/90 mmHg.

### 2.4 Statistical analysis

Continuous variables were expressed as median and interquartile range (IQR). The Lilliefors-corrected Kolmogorov-Smirnov test were used to assess normality. Categorical variables were expressed as number of subjects and percentage. Trend tests were performed to show the prevalence, awareness, treatment, and control of hypertension between 2005 and 2023. Prevalence was standardized using the age and sex of the general population in Geneva using the Geneva Census population data in 2023. The standardized prevalence was calculated using the function PostStratify. In the years 2005, 2006 and 2007, the annual average number of participants was lower than the expected 1000 participant per year because another cohort study was conducted in parallel using the same Bus Santé infrastructure. These years were then grouped in the analysis because of sample size. Trends in the prevalence of hypertension, awareness, treatment and control between 2005 and 2023 were further stratified by sex (male, female).

Comparisons between the prevalence, awareness, treatment, and control of hypertension between 2005 and 2014 and 2015–2023 were then performed. These two time periods were chosen to have two 10-year periods and also to reflect any potential changes due to the implementation of the new Swiss guidelines for hypertension in 2015 loosening treatment targets in hypertensive patients (Giezendanner et al., 2020). The comparisons between the two time periods were done by age group, sex, education level, income, smoking, BMI and medical history (diabetes, dyslipidemia, doctor visit in the past 12 months). For each variable, a *t*-test or ANOVA was performed on the standardized prevalence depending on binary or categorical variables respectively. Tests of distribution were performed showing a normal distribution for the standardized prevalence. The distribution of mean systolic and diastolic blood pressure was evaluated in individuals who were unaware of having hypertension, those who were aware and untreated, and those who were aware and treated.

Multivariable regression models using generalized linear models (svyglm function in R) were used to evaluate the associations between socio-economic determinants (age, sex, education, income), health determinants (smoking status, BMI, presence of diabetes, presence of cholesterol, doctor visit in the past 12 months) and the presence of hypertension, awareness, treatment in hypertensive individuals, and control in treated hypertensive individuals. The outcomes were binary and logit link function was used. Missing data were missing at random and complete case analysis was performed in the multivariable regression models. Data were separated into two periods: 2005–2014 and 2015–2023. Odds ratios were adjusted for age, sex and education level, measurements included the coefficient and 95 % confidence interval.

To look further into the determinants of hypertension prevalence, awareness, treatment and control, an intersectional approach considering age and sex was done to look at the differences in the prevalence for men and women per age group.

The figures were all computed using ggplot function found in the package GGally in R.

Statistical analyses were performed using R studio (R version 4.4.0).

## 3 Results

### 3.1 Baseline characteristics

A total of 11,278 participants were included in the analysis, of whom *n* = 5849 (50.8 %) were women. Overall mean age was 52.6 years old (SD, 10.9). Overall, 45.8 % of participants were between 35 and 49 years old, 36.9 % between 50 and 64 years old and 17.4 % 65 years and older. Most participants had a secondary (49.6 %) or tertiary (40.5 %) education level, and a middle to high income level (58.0 %). Among the participants, 47.8 % were never-smoker, 31.7 % were ex-smokers and 20.5 % were current smokers. Average BMI was 25.4 (SD, 4.4) kg/m^2^, with half of the participants falling in the normal weight category (BMI 18.5–24.9 kg/m2), and a third being overweight (BMI 25–29.9 kg/m2). When looking at medical history, 6.6 % of participants had diabetes, 30.2 % had dyslipidemia and 81.5 % had at least one doctor visit in the past 12 months. Table 1 summarizes the baseline characteristics of participants between 2005 and 2023.

**Table 1:** Distribution of baseline characteristics of adult participants of the Bus Sante study in Geneva, Switzerland, between 2005 and 2014 and 2015–2023 (*n* = 11,278).

		Time period	
Characteristic	Overall *N* = 11,278	2005–2014 *N* = 6402	2015–2023 *N* = 4876
**Age, median (IQR)**	51.4 (43.4, 61.3)	51.2 (43.2, 61.4)	51.6 (43.6, 61.2)
**Age group, N(%)**			
35–49	5160 (45.8)	2958 (46.2)	2202 (45.2)
50–64	4161 (36.9)	2353 (36.8)	1808 (37.1)
65+	1957 (17.4)	1091 (17.0)	866 (17.8)
**Sex, N(%)**			
Female	5757 (51.0)	3278 (51.2)	2479 (50.8)
Male	5521 (49.0)	3124 (48.8)	2397 (49.2)
**Education, N(%)**			
Primary	1094 (9.8)	717 (11.4)	377 (7.8)
Secondary	5519 (49.6)	3320 (52.7)	2199 (45.7)
Tertiary	4506 (40.5)	2265 (35.9)	2241 (46.5)
Missing	159	100	59
**Income, N(%)**			
Low	2187 (19.6)	1301 (20.3)	886 (18.7)
Middle-low	1845 (16.6)	1117 (17.4)	728 (15.4)
Middle-high	2185 (19.6)	1275 (19.9)	910 (19.2)
High	4271 (38.4)	2304 (36.0)	1967 (41.6)
Don't know/refuse	645 (5.8)	405 (6.3)	240 (5.1)
Missing	145	0	145
**BMI (kg/m**^**2**^**), median (IQR)**	24.9 (22.3, 27.8)	24.9 (22.3, 27.8)	24.7 (22.2, 27.8)
**BMI group, N(%)**			
Underweight; <18.5 kg/m^2^	248 (2.2)	141 (2.2)	107 (2.2)
Healthy; 18.5–24.9 kg/m^2^	5546 (49.2)	3109 (48.6)	2437 (50.0)
Overweight; 25–29.9 kg/m^2^	3881 (34.4)	2249 (35.1)	1632 (33.5)
Obesity; ≥30 kg/m^2^	1603 (14.2)	903 (14.1)	700 (14.4)
**Smoker status, N(%)**			
Never smokers	5354 (47.8)	2997 (47.0)	2357 (48.9)
Ex-smokers	3558 (31.7)	2035 (31.9)	1523 (31.6)
Current smokers	2295 (20.5)	1351 (21.2)	944 (19.6)
Missing	71	19	52
**Diabetes, N(%)**	739 (6.6)	415 (6.5)	324 (6.6)
**Dyslipidemia, N(%)**	3405 (30.2)	1999 (31.2)	1406 (28.9)
**Visited a doctor, N(%)**	9178 (81.5)	5202 (81.3)	3976 (81.8)

Values are N (%) unless otherwise specified. % may not add up to 100 % due to rounding. IQR: Interquartile range, BMI: Body mass index.

Education was defined as: primary (compulsory education), secondary (high school or apprenticeship), tertiary (university degree or above) based on the degree of education attained. Monthly household income was defined in Swiss francs (1 CHF = 1.13 USD on 13 March 2025) as: low (<5000), middle-low (5000–6999), middle-high (7000–9499), and high (≥9500), available response options “I don't know” and “I refuse to answer” were coded as missing.

### 3.2 Trends overtime

**Fig. 1:**
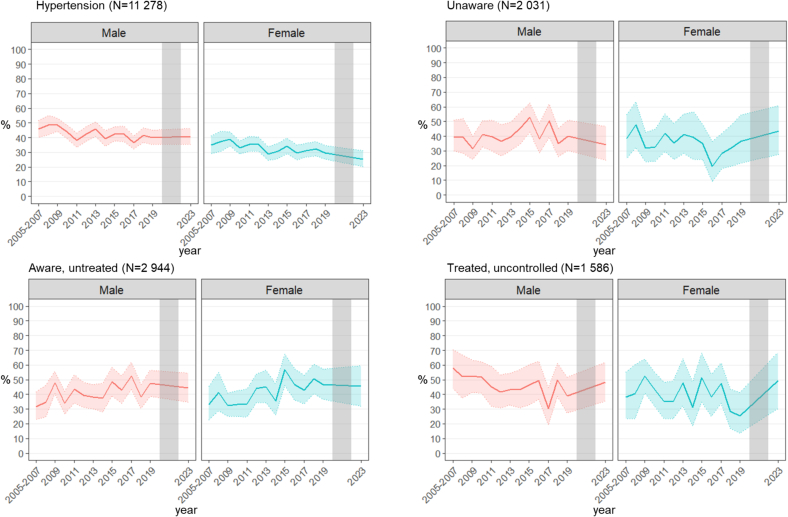
Distribution of age and sex-standardized prevalence, awareness, treatment and control of hypertension in adult participants of the Bus Sante study in Geneva, Switzerland, between 2005 and 2023 (n = 11,278).

**Fig. 2:**
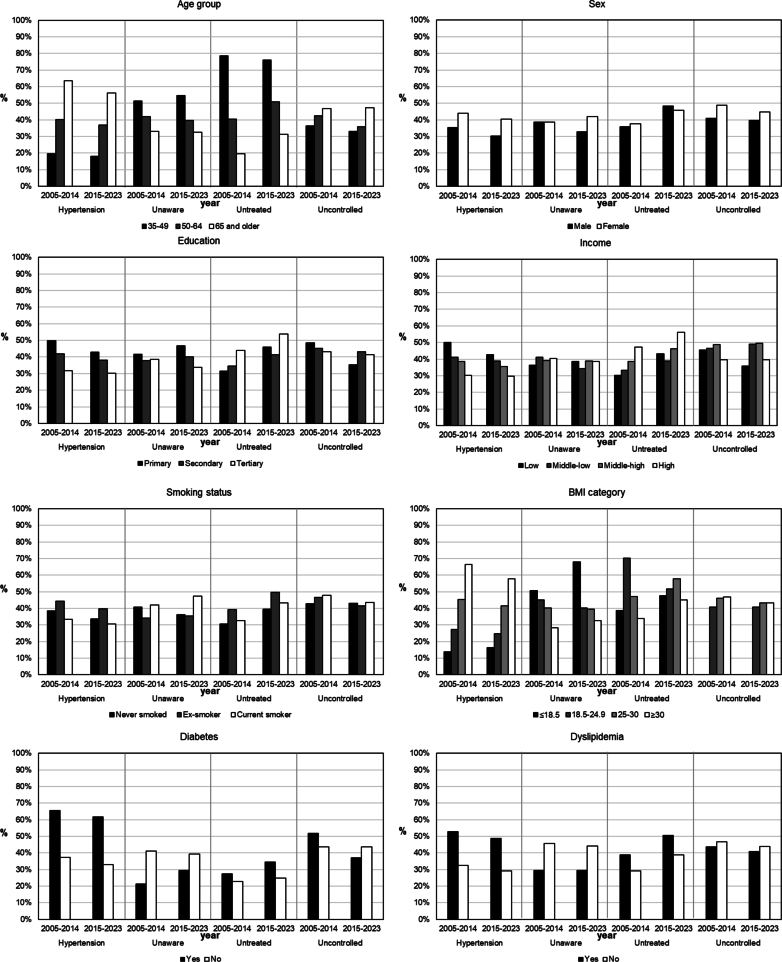
Distribution of hypertension prevalence, awareness, treatment and control by determinants, in adult participants of the Bus Sante study in Geneva, Switzerland, between 2005 and 2014 and 2015–2023 (n = 11,278).

Trends overtime showed a decreasing overall prevalence of hypertension. Fig. 1 shows the yearly trends overall and stratified by sex with the 95 % confidence interval. The standardized prevalence of hypertension decreased from 38.9 % to 35.2 % between 2005 and 2014 and 2015–2023 (*p* = 0.03), the proportion of individuals who were unaware of having hypertension did not significantly change overall. The proportion of individuals with hypertension who were untreated increased with time (37.7 % in 2005–2014 *versus* 46.8 % in 2015–2023, *p* < 0.001). However, trends showed less uncontrolled blood pressure among those who were treated (44.9 % between 2005 and 2014 *versus* 42.2 % between 2015 and 2023, *p* = 0.01). Trends were similar in men and women even though men had a higher prevalence of hypertension and were overall more unaware of having hypertension. Trends of being untreated increased upwards in both sexes with a higher proportion of women being untreated more recently (Δprev = +11.4 % in women *versus* Δprev = +6.9 % in men between 2005 and 2014 and 2015–2023), driven mostly by lack of treatment in older women. Men were also more likely to have uncontrolled blood pressure when treated and this remained true throughout the study period, although the proportion of uncontrolled hypertensive individuals decreased with time overall. Fig. 2 (and Supplementary Table 1) shows the difference in prevalence, awareness, treatment, and control of hypertension between 2005 and 2014 and 2015–2023. Supplementary Table 2 shows the distribution of these outcomes per age and sex groups.

### 3.3 Trends by socioeconomic and health determinants

Trends in hypertension prevalence decreased in older individuals (Δprev = −6.9 % in individuals 65 years and older), in individuals with a primary education level (Δprev = −6.1 %), and those with low income (Δprev = −6.1 %). Trends in lack of treatment in hypertensive individuals increased with time, and this was true for most categories except younger individuals (35–49 years old). Trends in hypertension control showed a decreasing prevalence of having uncontrolled hypertension Δprev = −2.6 %) overall. The prevalence of individuals with uncontrolled hypertension decreased with time especially in groups of individuals between 50 and 64 years old, with a primary education level, and with a low income (Δprev = −7.4 %; −13.2 % and − 9.8 % respectively). The prevalence of individuals with diabetes and uncontrolled hypertension also decreased with time (Δprev = −14.5 %).

### 3.4 Distribution of blood pressure measurements

**Fig. 3:**
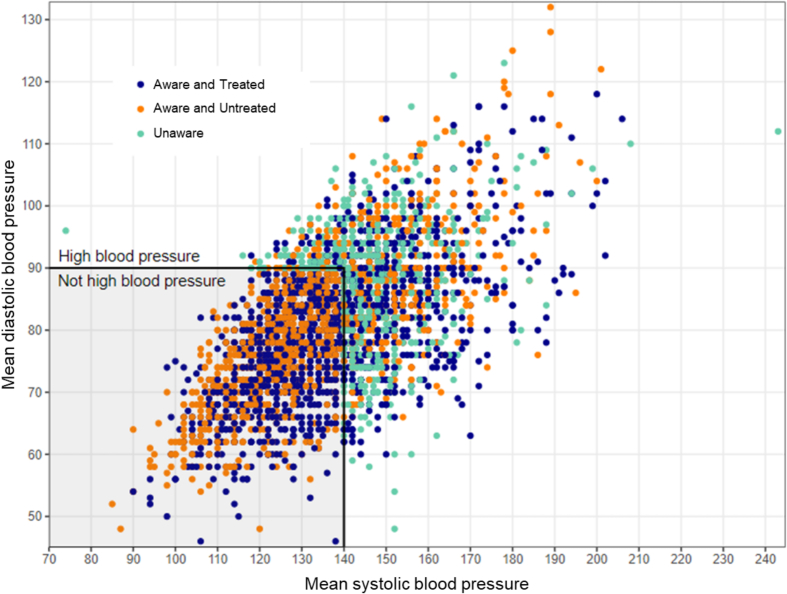
Distribution of mean blood pressure stratified by awareness and treatment in adult participants with hypertension of the Bus Sante study in Geneva, Switzerland between 2005 and 2023 (*n* = 3753).

The distribution of mean blood pressure depending on awareness and treatment show that 42.7 % of individuals who were aware and treated and 40.2 % of individuals who were aware and untreated had a mean systolic and/or diastolic blood pressure ≥140/90 mmHg (Fig. 3). Overall, mean systolic blood pressure was 116.8 mmHg in individuals aged 35–49 compared to 134.5 mmHg in individuals 65 years and older. The age-differential gradient was similar for individuals who were aware and treated, aware and untreated and unaware of having hypertension. Fig. 3 shows the distribution of mean blood pressure in the study population.

### 3.5 Determinants after adjusting for age, sex and education

After adjusting for age, sex, and education, older individuals had higher hypertension prevalence, were less likely to be unaware of having hypertension, were more likely to be treated and were more likely to have uncontrolled hypertension if treated. Male sex was associated with hypertension prevalence (aOR 1.8 [1.60–2.06]). Men were also more likely to be unaware of having hypertension (aOR 1.4 [1.02–1.99]). Individuals with a more advanced education level (secondary or tertiary) were less likely to have hypertension compared to those with a primary education level and were more likely to be aware of having hypertension. After adjusting for age, sex, and education, income levels were not associated with any of the four hypertension outcomes (prevalence, awareness, treatment or control). Being overweight or obese was associated with a higher prevalence of hypertension and less likelihood of being untreated (aOR 0.3 [0.24–0.48]). Diabetes was associated with hypertension prevalence and was not associated with awareness. However, individuals with diabetes became more likely to be treated for hypertension with time (aOR 0.4 [0.27–0.66]). Dyslipidemia was associated with the increased prevalence, awareness, and treatment of hypertension, however there was no association between dyslipidemia and hypertension control. Having a doctor visit in the past 12 months was associated with hypertension prevalence and treatment, increased awareness. However, having a doctor visit in the past 12 months was not associated with control of blood pressure.

Supplementary Table 3 summarizes the associations between the above determinants and the outcomes of hypertension prevalence, awareness, treatment and control.

## 4 Discussion

Trends of hypertension prevalence and control improved overall with time and in socioeconomically disadvantaged subgroups including individuals with a primary education or low income. However, hypertension awareness did not change with time, and increasingly more individuals are shown to be untreated when diagnosed with hypertension.

The prevalence of hypertension shows a downward trend especially in the past years and for women. These changes are new compared to our previous study on the same source population between 1999 and 2009 (Guessous et al., 2012). When looking into subgroups, the prevalence of hypertension decreased by 5 % in women and 3.7 % in men. Individuals with a primary level of education had the largest decrease in hypertension prevalence (Δprev = −6.1 %), as did those with a low-income level. This could be due to the fact that these groups were potentially better targeted in more recent years (Whelton et al., 2017), or that, as hypertension prevalence improves across all groups, these subgroups experienced greater differential improvements due to starting from a higher baseline. However, the overall prevalence of hypertension is still higher in individuals with a primary education level and low income. Our results are similar to previous studies and a meta-analysis showing the increased risk of hypertension associated with low socioeconomic status^25^. After adjustment, primary education level remains a determinant of hypertension in our study, whereas income level does not show an association with hypertension prevalence. This underscores the importance of enhancing support, guidance, and health education for individuals with lower education levels in global efforts to reduce hypertension prevalence.

Hypertension awareness did not improve overall in the recent years while it had previously significantly improved between 1999 and 2009 (Guessous et al., 2012). Gender-differential analysis shows differences in sex and age groups in this current study. Younger men are more unaware of having hypertension (58.7 % in 2015–2023 compared to 50.9 % in 2005–2014), while younger women became increasingly more aware of having hypertension (40.2 % unaware in 2015–2023 compared to 52.3 % unaware in 2005–2014). The increased awareness in younger women could be due to prenatal visits and screening (Chang et al., 2023), which is not seen in younger men. After adjustment, male sex was shown to be a risk factor for unawareness, and age a protective factor, along with tertiary education and a visit to the primary care physician in the past 12 months. These results are similar to other studies (Guo et al., 2012; de Mestral et al., 2024; Foti et al., 2019; Blok et al., 2022). However, they highlight the need to better target younger men to increase awareness and screening around hypertension, especially that men are more at risk of cardiovascular disease and hypertension.

Results showed an increase in the number of individuals who are untreated when diagnosed with hypertension. This could reflect the loosening in treatment targets following the 2015 Swiss guidelines for hypertension management. The revised guidelines in 2015 recommended a blood pressure target of less than 140/85 in hypertensive patients with diabetes and/or renal disease (Giezendanner et al., 2020). Results showed that older women seemed increasingly more untreated for hypertension compared to men and younger individuals (Δprev = +16.3 % between 2005 and 2014 and 2015–2023). The current guidelines for hypertension base the initiation of treatment on blood pressure measurements and do not differentiate treatment based on sex (Whelton et al., 2017). Sex-based differences with delay in diagnosis and treatment could be a major limitation in the proper management of chronic diseases in women (Rodriguez et al., 2024; DiGiacomo et al., 2015).

Hypertension control improved with time overall and in individuals with a primary education level and low income, reducing the gap between socioeconomic groups. The observed improvement in blood pressure control may reflect delayed benefits from increased awareness in the same source population between 1999 and 2009, during which blood pressure control had not significantly advanced (Guessous et al., 2012). However, closer analysis shows that older women with hypertension have worsening control between 2005 and 2014 and 2015–2023, and the age differential in blood pressure control is present in women but not in men. Older women with hypertension are then more at risk of having uncontrolled blood pressure, and as shown above, are increasingly more at risk of being untreated. Disparities in hypertension treatment and control highlight the need for better targeted interventions among women and especially older women.

Results show a trend towards better control of hypertension in individuals with diabetes, consistent with the 2017 guidelines for better management and control of blood pressure in diabetic patients. Visits to the primary care physician in the past 12 months were linked to treatment of hypertension, however, they were not associated with better control, suggesting potential inertia in hypertension management and the need to enhance adherence to treatment and to mitigate long-term consequences. A recent study showed the severe but avoidable long-term consequences of uncontrolled hypertension, encouraging physicians to increase awareness of the burden of hypertension, as well as offering training to patients on monitoring blood pressure levels and management (Mancia et al., 2023).

A key strength lies in its inclusion of data from 2023 after the COVID-19 pandemic. While results are still early to interpret with a smaller sample size in the post-pandemic compared to pre-pandemic period, the COVID-19 pandemic did not seem to disrupt overall trends of hypertension (Fig. 1). This 20-year population-based study also enables comparison a similar previous study conducted on the same source population from 1999 to 2009, providing a 24-year follow-up and including large sample sizes. Limitations include the use of self-reported data for treatment and previous diagnosis of hypertension; however, the objective measurements add a layer of scientific robustness to the study. Another limitation is a potential sample bias with a participation rate of 49 %. Efforts for recruitment (up to seven phone calls and two letters) are done to include as much as possible all participants who were randomly invited to participate. Standardization for age and sex of the general population and adjustments for age, sex and education in the regression models were used to improve the representativity of the sample and results. Another limitation includes the interruption of the study between 2020 and 2022 because of the COVID-19 pandemic, however, results here show a first look at the hypertension outcomes in this population post-pandemic. The study measures also remained identical pre- and post-pandemic, yet we cannot exclude any impact from the COVID-19 pandemic.

## 5 Conclusion

Trends of hypertension mostly improved with time, with decreasing prevalence, more awareness, more control and reduced socioeconomic disparities in hypertension prevalence and control. Older women are shown to be a population group where targeted interventions for increased blood pressure treatment and control are needed. Younger men should also benefit from more hypertension awareness, even though their blood pressure treatment and control seem adapted and improved with time. Primary care physicians should continue their efforts of screening for hypertension, educating individuals about treatment options and managing blood pressure to avoid long-term morbidity and mortality.

## Statement

There are no potential conflicts of interest related to this work. We confirm that the manuscript has been read and approved by all named authors and that there are no other persons who satisfied the criteria for authorship but are not listed. We further confirm that this work has been conducted with the ethical approval of the Cantonal Research Ethics Commission of Geneva, Switzerland, and that the approvals are acknowledged within the manuscript.

## CRediT authorship contribution statement

**Mayssam Nehme:** Writing – review & editing, Writing – original draft, Methodology, Investigation, Conceptualization. **Anshu Uppal:** Writing – review & editing, Visualization, Methodology, Formal analysis. **Ophelia Zimmerman:** Writing – review & editing, Visualization, Methodology, Formal analysis. **Julien Lamour:** Writing – review & editing, Data curation. **Shannon Mechoullam:** Writing – review & editing, Data curation. **Idris Guessous:** Writing – review & editing, Writing – original draft, Validation, Supervision, Project administration, Methodology, Conceptualization.

## Funding

This study was supported by the public entities of the Cantonal Office of Public Health and the 10.13039/501100006388Geneva University Hospitals. No private funding was used for this study.

## Declaration of competing interest

The authors declare that they have no known competing financial interests or personal relationships that could have appeared to influence the work reported in this paper.

## Data Availability

Data are available upon reasonable request made to corresponding author.

## Appendix A Supplementary data

Supplementary material: Supplementary data include the distribution of hypertension prevalence, awareness, treatment and control in adult participants of the Bus Sante study in Geneva, Switzerland between 2005 and 2023 (supplementary table 1), including an intersectional comparison by age and sex groups (supplementary table 2). The supplementary data also include the associations between determinants and hypertension prevalence, unawareness, lack of treatment and lack of control in adult participants of the Bus Sante study in Geneva, Switzerland (supplementary table 3) Supplementary material
