# Supplementary material for: Twenty years population-based trends in prevalence, awareness, treatment, and control of hypertension in Geneva, Switzerland
Source: Prev Med Rep. 2025 Apr 1;53:103055. doi: 10.1016/j.pmedr.2025.103055 (PMC11999646; doi:10.1016/j.pmedr.2025.103055)
Supplement: Supplementary material: Supplementary data include the distribution of hypertension prevalence, awareness, treatment and control in adult participants of the Bus Sante study in Geneva, Switzerland between 2005 and 2023 (supplementary table 1), including an intersectional comparison by age and sex gr [file mmc1.docx]

Supplementary table 1. Comparison of the distribution of hypertension prevalence, awareness, treatment and control in adult participants of the Bus Sante study in Geneva, Switzerland between 2005-2014 and 2015-2023 (n=11 278)

| **Hypertension** | | | | | | | | |  |
| --- | --- | --- | --- | --- | --- | --- | --- | --- | --- |
|  |  | **Prevalence** | | |  | **Unaware** | | | |
|  |  | 2005-2014 | 2015-2023 | Δprev |  | 2005-2014 | 2015-2023 | Δprev |  |
| Overall % [95%confidence interval] | | 38.9 [37.61, 40.27] | 35.2 [33.80, 36.70] | -3.7 |  | 38.9 [35.93, 41.88] | 38.1 [34.63, 41.69] | -0.8 |  |
| Age | 35-49 | 19.4 [18.01, 20.95] | 18.1 [16.56, 19.83] | -1.3 |  | 51.3 [45.20, 57.43] | 53.8 [45.82, 61.63] | 2.5 |  |
|  | 50-64 | 40.6 [38.55, 42.64] | 36.9 [34.69, 39.21] | -3.7 |  | 41.3 [37.05, 45.65] | 39.7 [34.63, 45.11] | -1.5 |  |
|  | 65 and older | 63.2 [60.14, 66.20] | 56.2 [52.83, 59.59] | -6.9 |  | 33.1 [28.50, 38.03] | 32.7 [27.53, 38.30] | -0.4 |  |
| Sex | Female | 34.4 [32.51, 36.29] | 30.4 [28.41, 32.49] | -3.9 |  | 38.6 [33.79, 43.59] | 32.8 [27.20, 39.04] | -5.7 |  |
|  | Male | 44.1 [42.23, 45.96] | 40.7 [38.66, 42.74] | -3.4 |  | 39.1 [35.48, 42.81] | 41.8 [37.53, 46.14] | 2.7 |  |
| Education | Primary | 49.3 [45.29, 53.26] | 43.2 [37.95, 48.60] | -6.1 |  | 41.5 [33.95, 49.56] | 46.7 [35.04, 58.66] | 5.1 |  |
|  | Secondary | 41.3 [39.48, 43.23] | 38.3 [36.12, 40.50] | -3.1 |  | 38.5 [34.62, 42.46] | 39.7 [34.90, 44.77] | 1.3 |  |
|  | Tertiary | 31.7 [29.65, 33.89] | 30.4 [28.35, 32.48] | -1.4 |  | 37.9 [32.41, 43.80] | 33.7 [28.37, 39.59] | -4.2 |  |
| Income | Low | 48.7 [45.70, 51.75] | 42.7 [39.20, 46.20] | -6.1 |  | 37.1 [31.47, 42.95] | 38.1 [30.64, 46.02] | 1.0 |  |
|  | Middle-low | 40.6 [37.45, 43.81] | 39.1 [35.28, 42.95] | -1.5 |  | 41.3 [34.40, 48.48] | 34.2 [26.49, 42.95] | -7.0 |  |
|  | Middle-high | 38.6 [35.72, 41.65] | 35.7 [32.41, 39.11] | -2.9 |  | 39.7 [33.45, 46.25] | 38.9 [31.49, 46.97] | -0.7 |  |
|  | High | 30.8 [28.77, 32.87] | 29.9 [27.83, 32.13] | -0.8 |  | 40.2 [34.68, 45.88] | 38.7 [32.88, 44.92] | -1.4 |  |
| Smoking status | Never smoked | 42.4 [37.07, 47.83] | 37.3 [30.76, 44.28] | -4.5 |  | 40.3 [35.83, 44.84] | 35.8 [30.80, 41.17] | -4.4 |  |
|  | Ex-smoker | 38.2 [36.23, 40.16] | 33.6 [31.60, 35.76] | -3.6 |  | 35.2 [30.64, 40.05] | 35.5 [30.07, 41.31] | 0.3 |  |
|  | Current smoker | 43.7 [41.34, 46.07] | 40.1 [37.47, 42.73] | -2.4 |  | 42.8 [35.83, 50.03] | 47.1 [38.24, 56.10] | 4.3 |  |
| BMI | Underweight; <18.5kg/m^2^ | 33.1 [30.29, 35.88] | 30.7 [27.61, 33.89] | 2.3 |  | 51.8 [21.60, 80.73] | 67.2 [28.67, 91.29] | 15.4 |  |
|  | Healthy; 18.5-24.9 kg/m^2^ | 14.1 [9.17, 21.06] | 16.4 [9.79, 26.15] | -1.9 |  | 45.2 [39.46, 51.02] | 40.1 [33.91, 46.66] | -5.1 |  |
|  | Overweight; 25-29.9 kg/m^2^ | 26.8 [25.01, 28.61] | 24.9 [23.01, 26.82] | -3.3 |  | 40.3 [35.94, 44.89] | 39.2 [33.85, 44.85] | -1.1 |  |
|  | Obesity; ≥ 30kg/m^2^ | 45.1 [42.79, 47.30] | 41.7 [39.16, 44.26] | -8.2 |  | 29.1 [24.23, 34.59] | 32.6 [26.46, 39.45] | 3.5 |  |
| Diabetes | Yes | 66.1 [62.68, 69.34] | 57.9 [54.03, 61.65] | -2.5 |  | 21.1 [14.58, 29.58] | 29.3 [20.25, 40.41] | 8.2 |  |
|  | No | 64.2 [59.19, 68.87] | 61.7 [56.07, 66.99] | -3.8 |  | 41.4 [38.22, 44.61] | 39.1 [35.44, 42.95] | -2.2 |  |
| Dyslipidemia | Yes | 37.1 [35.66, 38.40] | 33.2 [31.74, 34.71] | -3.1 |  | 29.2 [25.00, 33.74] | 29.2 [24.14, 34.81] | 0.01 |  |
|  | No | 51.9 [49.49, 54.26] | 48.8 [46.04, 51.58] | -3.1 |  | 46.3 [42.33, 50.25] | 44.0 [39.43, 48.68] | -2.3 |  |
| Doctor visit in the past 12 months | Yes | 32.4 [30.83, 33.93] | 29.2 [27.63, 30.93] | -4.0 |  | 34.7 [31.61, 38.02] | 33.3 [29.60, 37.14] | -1.5 |  |
|  | No | 41.1 [39.56, 42.54] | 37.1 [35.47, 38.71] | -2.3 |  | 61.6 [54.33, 68.40] | 60.2 [51.52, 68.21] | -1.4 |  |

| **Hypertension** | | | | | | | | | | |
| --- | --- | --- | --- | --- | --- | --- | --- | --- | --- | --- |
|  |  | **Untreated** | | |  | | **Uncontrolled** | | | |
|  |  | **2005-2014** | **2015-2023** | Δprev | |  | | **2005-2014** | **2015-2023** | Δprev |
| Overall % [95%confidence interval] | | 37.7 [35.34, 40.22] | 46.8 [43.93, 49.79] | 9.1 | |  | | 44.9 [41.47, 48.33] | 42.3 [38.17, 46.46] | -2.6 |
| Age | 35-49 | 78.5 [74.25, 82.17] | 75.9 [70.80, 80.39] | -2.5 | |  | | 36.3 [26.93, 46.83] | 32.9 [23.24, 44.34] | -3.4 |
|  | 50-64 | 40.5 [36.86, 44.24] | 50.8 [46.45, 55.09] | 10.3 | |  | | 43.2 [38.47, 48.11] | 35.9 [30.17, 41.98] | -7.4 |
|  | 65 and older | 19.6 [16.35, 23.29] | 31.3 [26.79, 36.17] | 11.7 | |  | | 46.7 [41.82, 51.58] | 47.2 [41.30, 53.26] | 0.6 |
| Sex | Female | 37.0 [33.38, 40.73] | 48.4 [43.92, 52.84] | 11.4 | |  | | 40.7 [35.66, 45.97] | 39.5 [33.23, 46.07] | -1.2 |
|  | Male | 38.4 [35.27, 41.74] | 45.4 [41.63, 49.26] | 6.9 | |  | | 48.8 [44.30, 53.30] | 44.8 [39.49, 50.15] | -4.1 |
| Education | Primary | 32.6 [26.58, 39.29] | 45.7 [36.27, 55.48] | 13.1 | |  | | 48.4 [39.65, 57.27] | 35.2 [23.97, 48.42] | -13.2 |
|  | Secondary | 35.4 [32.25, 38.79] | 41.3 [37.26, 45.51] | 5.9 | |  | | 45.0 [40.44, 49.60] | 43.0 [37.42, 48.80] | -2.0 |
|  | Tertiary | 44.9 [40.40, 49.46] | 53.6 [49.02, 58.21] | 8.7 | |  | | 42.7 [36.33, 49.29] | 41.4 [34.62, 48.55] | -1.3 |
| Income | Low | 31.2 [26.74, 36.09] | 43.1 [36.99, 49.28] | 11.8 | |  | | 45.7 [39.39, 52.14] | 43.0 [36.90, 49.41] | -9.8 |
|  | Middle-low | 34.4 [29.14, 40.17] | 38.7 [32.05, 45.87] | 4.3 | |  | | 46.2 [38.65, 53.99] | 41.4 [35.07, 48.12] | 2.7 |
|  | Middle-high | 39.6 [34.15, 45.30] | 45.9 [39.28, 52.68] | 6.3 | |  | | 48.3 [40.33, 56.42] | 43.3 [33.26, 53.94] | 1.1 |
|  | High | 47.4 [42.85, 51.94] | 56.0 [51.07, 60.79] | 8.6 | |  | | 40.0 [33.63, 46.65] | 12.7 [1.54, 57.66] | -0.5 |
| Smoking status | Never smoked | 40.2 [36.56, 44.03] | 49.4 [45.08, 53.75] | 9.2 | |  | | 42.8 [37.72, 48.11] | 40.7 [32.92, 48.98] | 0.2 |
|  | Ex-smoker | 33.8 [30.15, 37.77] | 43.1 [38.41, 47.95] | 9.3 | |  | | 46.6 [41.28, 52.01] | 43.2 [36.88, 49.79] | -5.2 |
|  | Current smoker | 39.9 [34.16, 45.90] | 47.5 [40.43, 54.59] | 7.6 | |  | | 46.7 [38.22, 55.34] | 43.2 [36.04, 50.63] | -3.4 |
| BMI | Underweight; <18.5kg/m^2^ | 73.7 [43.67, 91.05] | 52.1 [23.91, 78.94] | -21.7 | |  | | [NA, NA] | 37.1 [28.53, 46.64] | -87.3 |
|  | Healthy; 18.5-24.9 kg/m^2^ | 48.5 [43.94, 53.09] | 57.6 [52.48, 62.62] | 9.1 | |  | | 40.1 [33.58, 47.03] | 43.5 [38.90, 48.18] | 0.6 |
|  | Overweight; 25-29.9 kg/m^2^ | 35.1 [31.42, 38.95] | 45.2 [40.56, 49.80] | 10.1 | |  | | 46.3 [40.98, 51.44] | 40.7 [35.19, 46.54] | -2.9 |
|  | Obesity; ≥ 30kg/m^2^ | 28.1 [23.98, 32.55] | 34.2 [29.09, 39.73] | 6.1 | |  | | 46.6 [40.59, 52.79] | 44.0 [38.01, 50.13] | -3.5 |
| Diabetes | Yes | 23.6 [18.13, 30.11] | 24.8 [18.43, 32.50] | 1.2 | |  | | 51.6 [43.26, 59.84] | 42.4 [38.15, 46.67] | -14.5 |
|  | No | 39.9 [37.31, 42.62] | 50.3 [47.14, 53.46] | 10.4 | |  | | 43.5 [39.76, 47.26] | 42.2 [25.90, 60.46] | 0.01 |
| Dyslipidemia | Yes | 30.6 [27.33, 34.04] | 38.6 [34.43, 42.91] | 8.1 | |  | | 43.8 [39.19, 48.55] | 43.0 [36.90, 49.41] | -3.1 |
|  | No | 44.6 [41.14, 48.10] | 53.9 [49.87, 57.83] | 9.3 | |  | | 46.2 [41.21, 51.25] | 41.4 [35.07, 48.12] | -2.2 |
| Doctor visit in the past 12 months | Yes | 34.9 [32.46, 37.50] | 44.1 [40.98, 47.11] | 9.1 | |  | | 45.0 [41.49, 48.56] | 43.3 [33.26, 53.94] | -2.6 |
|  | No | 65.6 [57.43, 72.91] | 75.5 [66.68, 82.53] | 9.9 | |  | | 42.5 [29.29, 56.93] | 12.7 [1.54, 57.66] | -0.3 |

BMI: Body mass index.

Supplementary table 1 shows hypertension prevalence, awareness, treatment and control between the two time periods (2005-2014 and 2015-2023). The results show the percentages of individuals with hypertension, individuals who have hypertension but are unaware (unawareness), individuals who are aware to have hypertension but are not treated (untreated), and individuals who have hypertension, are treated but are uncontrolled (blood pressure measurement ≥140/90mmHg). Education was defined as: primary (compulsory education), secondary (high school or apprenticeship), tertiary (university degree or above) based on the degree of education attained. Monthly household income was defined in Swiss francs (1 CHF = 1.13 USD on 13 March 2025) as: low (< 5000), middle-low (5000–6999), middle-high (7000–9499), and high (≥ 9500), available response options “I don’t know” and “I refuse to answer” were coded as missing.

Supplementary Table 2. Comparison of the distribution of the prevalence, awareness, treatment and control between 2005-2014 and 2015-2023 per age and sex groups in adult participants of the Bus Sante study in Geneva, Switzerland (n=11 278)

|  |  | **Prevalence** | |  | **Unaware** | | |  |
| --- | --- | --- | --- | --- | --- | --- | --- | --- |
|  |  | 2005-2014 | 2015-2023 |  | 2005-2014 | 2015-2023 | |  |
|  |  | % [95%CI] | % [95%CI] |  | % [95%CI] | % [95%CI] | |  |
| Male | 30-49 | 23.4 [21.28, 25.75] | 23.2 [20.75, 25.88] |  | 50.9 [43.57, 58.27] | 57.7 [48.47, 66.36] | |  |
|  | 50-64 | 48.1 [45.16, 51.12] | 43.6 [40.28, 46.90] |  | 42.0 [36.65, 47.46] | 42.1 [35.90, 48.67] | |  |
|  | 65 and older | 70.5 [66.28, 74.34] | 63.7 [59.17, 68.11] |  | 31.0 [25.19, 37.41] | 34.3 [27.62, 41.75] | |  |
| Female | 30-49 | 15.6 [13.81, 17.63] | 13.3 [11.43, 15.43] |  | 52.2 [41.21, 62.97] | 40.3 [25.43, 57.14] | |  |
|  | 50-64 | 33.4 [30.75, 36.20] | 30.6 [27.69, 33.74] |  | 40.1 [33.26, 47.39] | 34.8 [26.29, 44.38] | |  |
|  | 65 and older | 57.9 [53.51, 62.11] | 50.7 [45.88, 55.48] |  | 35.1 [28.22, 42.63] | 31.2 [23.72, 39.76] | |  |
|  |  | **Untreated** | |  | **Uncontrolled** | | | |
|  |  | 2005-2014 | 2015-2023 |  | 2005-2014 | | 2015-2023 | |
|  |  | % [95%CI] | % [95%CI] |  | % [95%CI] | | % [95%CI] | |
| Male | 30-49 | 76.2 [70.26, 81.30] | 75.4 [68.43, 81.24] |  | 44.4 [31.68, 57.84] | | 42.4 [28.66, 57.48] | |
|  | 50-64 | 40.5 [35.64, 45.56] | 50.2 [44.30, 56.04] |  | 48.4 [41.86, 54.96] | | 42.6 [34.64, 51.04] | |
|  | 65 and older | 19.2 [14.98, 24.35] | 25.8 [20.41, 31.98] |  | 49.6 [43.12, 56.16] | | 46.4 [38.89, 54.10] | |
| Female | 30-49 | 81.3 [75.11, 86.25] | 76.6 [68.61, 83.08] |  | 23.4 [12.25, 39.94] | | 19.8 [9.57, 36.43] | |
|  | 50-64 | 40.5 [35.09, 46.13] | 51.5 [45.09, 57.79] |  | 36.7 [29.98, 43.96] | | 27.9 [20.37, 36.89] | |
|  | 65 and older | 19.9 [15.32, 25.47] | 36.3 [29.41, 43.71] |  | 43.9 [36.90, 51.18] | | 48.1 [38.93, 57.42] | |

CI: Confidence interval. Supplementary table 2 shows hypertension prevalence, awareness, treatment and control between the two time periods (2005-2014 and 2015-2023) stratified by age and sex. The results show the percentages of individuals with hypertension, individuals who have hypertension but are unaware (unawareness), individuals who are aware to have hypertension but are not treated (untreated), and individuals who have hypertension, are treated but are uncontrolled (blood pressure measurement ≥140/90mmHg). These percentages are stratified by age (30-49; 50-64; 65 and older) and sex (male; female) for each category.

Supplementary Table 3. Associations between determinants and hypertension prevalence, unawareness, lack of treatment and lack of control in adult participants of the Bus Sante study in Geneva, Switzerland, over the time periods 2005-2014 and 2015-2023 (n=11 278)

|  |  |  | | Hypertension | | | |  | | Unaware | | | |  | | Untreated | | | |  | | | Uncontrolled | | | | |  |
| --- | --- | --- | --- | --- | --- | --- | --- | --- | --- | --- | --- | --- | --- | --- | --- | --- | --- | --- | --- | --- | --- | --- | --- | --- | --- | --- | --- | --- |
|  |  |  | | 2005-2014 | | 2015-2023 | |  | | 2005-2014 | | 2015-2023 | |  | | 2005-2014 | | 2015-2023 | | |  | | | 2005-2014 | | 2015-2023 | |  |
| Age | 35-49 |  | | Ref | | Ref | |  | | Ref | | Ref | |  | | Ref | | Ref | | |  | | | Ref | | Ref | |  |
|  | 50-64 |  | | 2.8 [2.47, 3.21]* | | 2.6 [2.20, 2.97]* | |  | | 07 [0.50, 0.92]* | | 0.6 [0.38, 0.83]* | |  | | 0.2 [0.14, 0.25]* | | 0.3 [0.24, 0.46]* | | |  | | | 1.3 [0.79, 2.05] | | 1.1 [0.63, 1.88] | |  |
|  | 65 and older |  | | 7.6 [6.47, 9.00]* | | 5.9 [4.94, 7.07]* | |  | | 0.5 [0.33, 0.64]* | | 0.4 [0.29, 0.65]* | |  | | 0.1 [0.05, 0.10]* | | 0.1 [0.10, 0.20]* | | |  | | | 1.6 [0.99, 2.58] | | 1.8 [1.06, 3.11]* | |  |
| Sex | Female |  | | Ref | | Ref | |  | | Ref | | Ref | |  | | Ref | | Ref | | |  | | | Ref | | Ref | |  |
|  | Male |  | | 1.8 [1.60, 2.06]* | | 1.9 [1.63, 2.14]* | |  | | 0.9 [0.69, 1.20] | | 1.4 [1.02, 1.99]* | |  | | 0.9 [0.70, 1.17] | | 0.7 [0.56, 0.94]* | | |  | | | 1.5 [1.08, 1.98]* | | 1.3 [0.95, 1.93] | |  |
| Education | Primary |  | | Ref | | Ref | |  | | Ref | | Ref | |  | | Ref | | Ref | | |  | | | Ref | | Ref | |  |
|  | Secondary |  | | 0.7 [0.56, 0.83]* | | 0.7 [0.59, 0.97]* | |  | | 0.9 [0.58, 1.28] | | 0.8 [0.44, 1.34] | |  | | 1.2 [0.82, 1.79] | | 0.9 [0.54, 1.46] | | |  | | | 0.8 [0.54, 1.30] | | 1.2 [0.66, 2.21] | |  |
|  | Tertiary |  | | 0.5 [0.40, 0.61]* | | 0.6 [0.46, 0.76]* | |  | | 0.9 [0.56, 1.35] | | 0.6 [0.31, 0.98]* | |  | | 1.5 [0.99, 2.26] | | 1.5 [0.88, 2.44] | | |  | | | 0.8 [0.47, 1.23] | | 1.1 [0.60, 2.12] | |  |
| Income | Low |  | | Ref | | Ref | |  | | Ref | | Ref | |  | | Ref | | Ref | | |  | | | Ref | | Ref | |  |
|  | Middle-low |  | | 0.8 [0.68, 1.03] | | 1.1 [0.80, 1.28] | |  | | 1.2 [0.81, 1.84] | | 0.8 [0.51, 1.39] | |  | | 0.9 [0.63, 1.42] | | 0.7 [0.49, 1.12] | | |  | | | 1.1 [0.70, 1.66] | | 1.7 [1.01, 2.97]* | |  |
|  | Middle-high |  | | 0.9 [0.75, 1.13] | | 0.9 (0.7-1.09) | |  | | 1.1 [0.72, 1.58] | | 1.0 [0.61, 1.62] | |  | | 1.0 [0.67, 1.48] | | 0.9 [0.63, 1.41] | | |  | | | 1.2 [0.74, 1.86] | | 1.8 [1.03, 3.05]* | |  |
|  | High |  | | 0.7 [0.61, 0.90]* | | 0.9 [0.71, 1.06] | |  | | 1.1 [0.72, 1.62] | | 1.0 [0.63, 1.63] | |  | | 1.1 [0.75, 1.57] | | 1.2 [0.81, 1.76] | | |  | | | 0.8 [0.49, 1.23] | | 1.1 [0.67, 1.95] | |  |
| Smoking status | Never smoked |  | | Ref | | Ref | |  | | Ref | | Ref | |  | | Ref | | Ref | | |  | | | Ref | | Ref | |  |
|  | Ex smoker |  | | 1.1 [0.92, 1.24] | | 1.1 [0.93, 1.27] | |  | | 0.7 [0.55, 1.02] | | 0.9 [0.66, 1.33] | |  | | 0.8 [0.62, 1.09] | | 0.9 [0.67, 1.20] | | |  | | | 1.1 [0.79, 1.55] | | 0.8 [0.56, 1.23] | |  |
|  | Current smoker |  | | 0.9 [0.75, 1.06] | | 0.9 [0.78, 1.14] | |  | | 1.0 [0.65, 1.41] | | 1.5 [0.94, 2.27] | |  | | 0.8 [0.56, 1.10] | | 0.8 [0.56, 1.14] | | |  | | | 1.2 [0.77, 1.90] | | 1.1 [0.62, 1.78] | |  |
| Body-mass index (BMI) | Normal |  | | Ref | | Ref | |  | | Ref | | Ref | |  | | Ref | | Ref | | |  | | | Ref | | Ref | |  |
|  | Underweight |  | | 0.5 [0.27, 0.92] | | 0.6 [0.35, 1.19] | |  | | 1.3 [0.31, 5.35] | | 3.5 [0.62, 20.31] | |  | | 1.6 [0.56, 4.55] | | 0.8 [0.25, 2.88] | | |  | | | NA | | NA | |  |
|  | Overweight |  | | 1.7 [1.49, 2.01]* | | 1.7 [1.49, 2.04]* | |  | | 0.8 [0.56, 1.10] | | 0.8 [0.55, 1.16] | |  | | 0.6 [0.46, 0.84]* | | 0.7 [0.48, 0.90]* | | |  | | | 1.1 [0.77, 1.69] | | 1.1 [0.68, 1.68] | |  |
|  | Obese |  | | 4.2 [3.41, 5.16]* | | 3.7 [3.06, 4.59]* | |  | | 0.4 [0.29, 0.62]* | | 0.5 [0.36, 0.84]* | |  | | 0.4 [0.29, 0.59]* | | 0.3 [0.24, 0.48]* | | |  | | | 1.2 [0.78, 1.78] | | 1.2 [0.77, 1.97] | |  |
| Diabetes | | |  | | 2.1 [1.58, 2.65]* | | 2.3 [1.79, 3.01]* | |  | | 0.4 [0.24, 0.69]* | | 0.7 [0.41, 1.21] | |  | | 0.6 [0.38, 0.92]* | | 0.4 [0.27, 0.66]* | | |  | | | 1.3 [0.85, 1.91] | | 0.6 [0.41, 1.01] | |
| Dyslipidemia | | |  | | 1.5 [1.33, 1.76]* | | 1.7 [1.49, 1.99]* | |  | | 0.5 [0.38, 0.69]* | | 0.5 [0.37, 0.71]* | |  | | 0.7 [0.54, 0.91]* | | 0.6 [0.50, 0.85]* | | |  | | | 0.8 [0.60, 1.11] | | 0.8 [0.57, 1.17] | |
| Doctor visit in the  past 12 months | | |  | | 1.4 [1.21, 1.71]* | | 1.6 [1.30, 1.91]* | |  | | 0.3 [0.24, 0.51]* | | 0.4 [0.26, 0.58]* | |  | | 0.3 [0.21, 0.53]* | | 0.3 [0.18, 0.48]* | | |  | | | 1.2 [0.63, 2.36] | | 0.9 [0.42, 1.95] | |
| *: statistically significant with p-value <0.05.  NA: not applicable due to small sample size. Ref: reference  Supplementary table 3 shows hypertension prevalence, awareness, treatment and control between the two time periods (2005-2014 and 2015-2023) stratified by age and sex. The results show the percentages of individuals with hypertension, individuals who have hypertension but are unaware (unawareness), individuals who are aware to have hypertension but are not treated (untreated), and individuals who have hypertension, are treated but are uncontrolled (blood pressure measurement ≥140/90mmHg). Multivariable regression models using generalized linear models (svyglm function in R) were used to evaluate the associations between socio-economic determinants and the presence of hypertension, awareness, treatment in hypertensive individuals, and control in treated hypertensive individuals. Adjusted odds ratios were adjusted for age, sex, an education level. Results show adjusted odds ratios with 95% confidence interval. Education was defined as: primary (compulsory education), secondary (high school or apprenticeship), tertiary (university degree or above) based on the degree of education attained. Monthly household income was defined in Swiss francs (1 CHF = 1.13 USD on 13 March 2025) as: low (< 5000), middle-low (5000–6999), middle-high (7000–9499), and high (≥ 9500), available response options “I don’t know” and “I refuse to answer” were coded as missing. BMI groups were defined as: underweight (<18.5 kg/m2), healthy (18.5 to less than 25 kg/m2), overweight (25 to less than 30 kg/m2), obesity (30 kg/m2 or greater). | | | | | | | | | | | | | | | | | | | | | | | | | | | | |
